# Sun-Exposed vs. Non-Sun-Exposed Areas: Epidemiology and Pathogenesis of Non-Metastatic Merkel Cell Carcinoma

**DOI:** 10.3390/diagnostics16050818

**Published:** 2026-03-09

**Authors:** Alexander Yakobson, Ronen Brenner, Hanna T. Frumin Edri, Anna Ievko, Sofiia Turaieva, Tanzilya Tairov, Ilia Berezhnov, Shlomit Fenig, Eyal Fenig, Tomer Ziv-Baran, Sabri El-Saied, Walid Shalata

**Affiliations:** 1 The Legacy Heritage Cancer Center and Dr. Larry Norton Institute, Soroka Medical Center, Beer Sheva 84105, Israel; 2 Medical School for International Health, Faculty of Health Sciences, Ben Gurion University of the Negev, Beer Sheva 84105, Israel; 3 Oncology Institute, Edith Wolfson Medical Center, Holon 58220, Israel; 4 Faculty of Medicine, Tel Aviv University, Tel Aviv 69978, Israel; 5 Institute of Oncology, Kaplan Medical Center, Faculty of Medicine, Hebrew University, Jerusalem 9190500, Israel; 6 Institute of Oncology, Davidoff Center, Rabin Medical Center, Beilinson Hospital, Petah Tikva 4941492, Israel; 7 Department of Epidemiology and Preventive Medicine, School of Public Health, Faculty of Medicine, Tel Aviv University, Tel Aviv 6997801, Israel

**Keywords:** Merkel cell carcinoma, sunlight exposure, survival analysis, long-term outcomes

## Abstract

**Background:** Merkel cell carcinoma (MCC) is a rare and aggressive cutaneous neuroendocrine malignancy. The prognostic impact of sun exposure at the primary tumor site in localized and locally advanced MCC remains incompletely defined. We aimed to compare clinicopathologic characteristics and survival outcomes between sun-exposed and non-sun-exposed MCC in a large, multi-center Israeli cohort. **Methods:** We retrospectively identified 249 patients diagnosed with localized or locally advanced MCC between January 1985 and December 2020. Of these, 225 patients met eligibility criteria and were included in the analysis: 142 with sun-exposed primary tumors (cohort A) and 83 with non-sun-exposed tumors (cohort B). Baseline characteristics included age, sex, tumor size, lymph node (LN) involvement at diagnosis, disease-free survival (DFS), and overall survival (OS). **Results:** Median age at diagnosis was similar between cohorts (~73 years), with a male predominance in both groups. LN involvement was significantly more frequent in non-sun-exposed tumors compared with sun-exposed tumors (57.0% vs. 30.0%, *p* < 0.001), while tumor size distribution did not differ significantly. Median DFS was numerically longer in sun-exposed patients (58.0 vs. 47.8 months, *p* ≈ 0.18), whereas median OS favored non-sun-exposed patients (89.7 vs. 79.7 months, *p* ≈ 0.21), though neither difference reached statistical significance overall. Females demonstrated longer DFS and OS than males across both cohorts. Among LN-negative patients, non-sun-exposed tumors were associated with significantly improved OS (105.9 vs. 91.4 months, *p* ≈ 0.03), particularly in males. Primary tumor size further stratified outcomes: non-sun-exposed patients had significantly superior OS for tumors <2 cm and both improved DFS and OS for tumors ≥2 cm. **Conclusions:** In this large real-world MCC cohort, sun exposure status was associated with distinct patterns of nodal involvement and survival in clinically relevant subgroups. Non-sun-exposed MCC demonstrated favorable survival outcomes, particularly in LN-negative disease and across tumor size categories, suggesting underlying biological differences that merit further investigation.

## 1. Introduction

Toker initially coined the term “trabecular carcinoma of the skin” in 1972 to describe Merkel cell carcinoma (MCC) based on its histopathological characteristics [1]. This rare neuroendocrine carcinoma of the skin saw enhanced diagnostic accuracy with the introduction of immunohistochemistry staining for cytokeratin 20 positivity and TTF-1 negativity, markers commonly shared among neuroendocrine tumors [1,2].

MCC, accounting for less than 1% of all skin malignancies, has an incidence rate of 0.5–0.7 cases per 100,000 person-years among adult patients over 75 years old. In contrast, the incidence rate for younger patients (ages 40 to 44) is 0.1 per 100,000 person-years. This cancer type exhibits a notable tendency toward localized recurrence and metastasis. It stands as the second deadliest form of skin cancer, trailing only melanoma in terms of mortality rates (1–3). The tumor typically appears as rapidly growing lesions, which it is frequently doubles in size within a span of 1 to 3 months, and may exhibit ulceration, often presenting as painless, firm nodules, or plaque-like, flesh-colored to red-violet lesions, sometimes accompanied by necrosis [1,2,3,4].

MCC tends to predominantly affect men, showing a ratio of 2:1 in comparison to women. Its primary oncogenic factors involve the presence of Merkel cell polyomavirus (MCPyV), identified in roughly 80% of cases, along with long exposure to ultraviolet radiation with the most affected areas are the head and neck, followed by the extremities [5,6,7,8,9]. Typically, those diagnosed are elderly (75 to 79 years), and are predominantly from a white population. Approximately 10% of cases are linked to immunosuppression due to factors such as transplantation, HIV infection, or hematologic malignancies, leading to a more challenging prognosis [1,2,3,4,7,8,9,10]. The diagnosis of MCC requires histopathological confirmation obtained through skin biopsy. Following diagnosis, sentinel lymph node evaluation is recommended for all clinically node-negative patients, given the high rate of occult nodal metastases and the typically aggressive clinical course of the disease. Management of localized MCC generally consists of wide local excision, often combined with adjuvant radiotherapy depending on risk factors and pathological findings [11]. Around 23% to 45% of patients diagnosed with MCC across all sites exhibit pathologic involvement of regional LN, while 8% show distant metastases upon diagnosis. The risk of recurrence varies based on the stage: from 20% for stage I disease to 75% for stage III disease. Recurrence typically occurs within a median time of 7 to 9 months, with 80% to 90% of cases recurring within the initial 2 years. The 5-year overall survival estimates stand at nearly 50% for local disease, approximately 35% for regional metastatic disease, and roughly 13% for distant metastatic disease [1,3,4,5,12,13]. In uncommon instances, patients may display clinically positive nodal disease along with distinct immunohistochemical markers of MCC without a detectable primary tumor. The reported occurrence of MCC with an unknown primary tumor origin in extensive studies falls within the range of 8% to 19% [14]. Despite the well-established role of ultraviolet (UV) radiation in the pathogenesis of MCC, important gaps remain in understanding whether tumors arising in sun-exposed versus non-sun-exposed skin represent biologically and clinically distinct entities. While prior studies have demonstrated differences in mutational burden, UV-signature mutations, and the relative contribution of MCPyV between chronically sun-exposed and less-exposed anatomical sites, the clinical implications of tumor location remain insufficiently characterized. In particular, limited data are available regarding potential differences in patterns of lymph node involvement, recurrence risk, and long-term survival outcomes according to sun exposure status. Most published series focus primarily on viral status or overall prognostic factors without specifically comparing clinically meaningful subgroups based on anatomical sun exposure. Therefore, whether primary tumor location in sun-exposed versus non-sun-exposed skin translates into distinct nodal behavior or survival outcomes remains unclear. Addressing this gap may improve risk stratification and provide insights into the biological heterogeneity of MCC [8,15,16].

In this article, our goal was to explore potential discrepancies in OS and DFS concerning different body areas affected by MCC, particularly comparing the impact between sun-exposed areas and those with limited or non-sunlight exposure.

## 2. Materials and Methods

### 2.1. Patient Selection

A non-interventional observational, retrospective study managed across multiple institutions. The study enrolled a cohort consisting of all patients diagnosed with localized or locally advanced MCC and treated with radiotherapy, chemotherapy, or a combination of both (depending on stage and lymph node involvement) at least a year before enrollment.

### 2.2. Patient Data Collection

**Figure 1:**
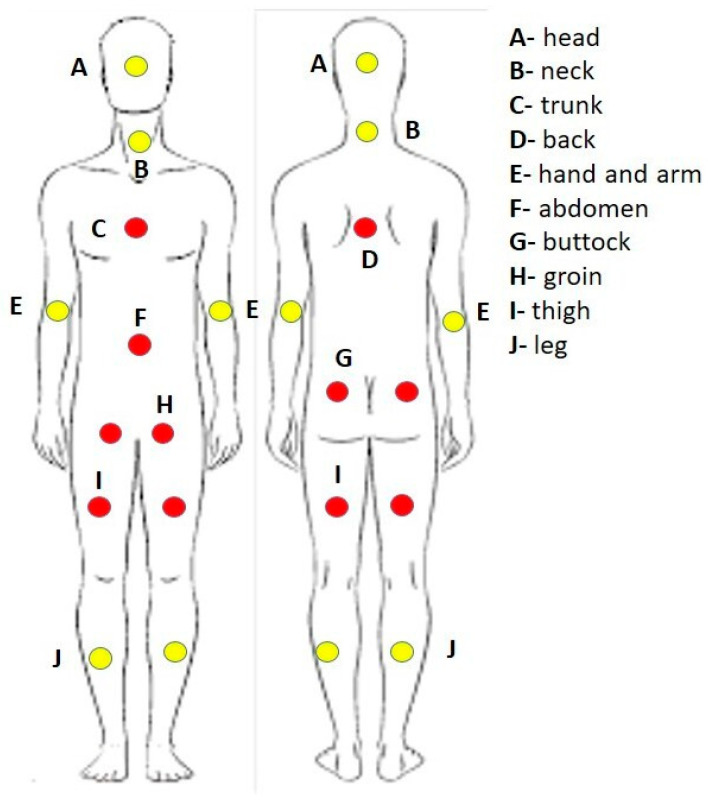
The classification of the sun-exposed areas (yellow points) and the limited or no sunlight exposure (red points).

Collected data of patients were treatment details, LN involvement, OS, mortality date, DFS durations (disease relapse date), the primary tumor sites were classified into two categories: sun-exposed areas and those with limited or no sunlight exposure (Figure 1), the date of the last follow-up, which was conducted up until November 2023.

### 2.3. Pre-Treatment Evaluations and Monitoring

Before treatment initiation, patients underwent disease staging through total body computed tomography (CT) scans or fluorodeoxyglucose (FDG) positron emission tomography computed tomography (PET-CT). Follow-up radiologic reevaluations (CT or FDG-PET-CT) (Armonk, New York, NY, United States) depending on the oncologist’s opinion or assessment at that time, all patients were evaluated through sentinel lymph node biopsy for checking LN status.

### 2.4. Inclusion Criteria

Patients meeting the following criteria were included:

Age requirement: Patients aged 18 years or older.

Confirmed diagnosis: Histologically confirmed Merkel cell carcinoma (MCC), any T or N with M0.

Initial treatment: Initiation of treatment involving radiotherapy, chemotherapy, or a combination based on stage and lymph node involvement.

No prior therapy: Absence of prior radiotherapy or systemic therapy for local or locally advanced disease.

Treatment source: Treatment administered at one of the participating medical centers or complete follow-up records available.

Multidisciplinary evaluation: Each study patient underwent assessment by a multidisciplinary medical team upon admission to the oncology institutes, following standard protocols.

This team comprised a pathologist, dermatologist, medical and radiation oncologists, plastic surgeon, radiologist and nuclear physician. Discussions were guided by the patient’s pathological and imaging status, as well as their performance status. A primary physician was assigned to oversee each patient’s treatment.

Patients diagnosed with local or locally advanced disease were primarily managed by radiation or medical oncologists, adhering to the National Comprehensive Cancer Network (NCCN) recommendations [14].

### 2.5. Exclusion Criteria

Patients that were excluded were those with metastatic disease, previous prior radiotherapy or systemic therapy, a non-MCC diagnosis, and who did not have DFS or OS. All patients presented with MCC manifesting as nodules rather than subcutaneous plaques. Patients with unknown primary tumors presenting solely with lymph node involvement were excluded from the analysis.

### 2.6. Statistical Analysis

Categorical variables were summarized as frequencies and percentages. The distribution of continuous variables was evaluated using histograms and Q-Q plots. Continuous variables were reported as the median and interquartile range (IQR). An independent samples t-test and a Mann–Whitney test were used to compare continuous variables between treatment groups, while categorical variables were compared using the Chi-square test or Fisher’s exact test. The reverse censoring method was used to evaluate the median length of follow-up. Survival analysis was conducted using Kaplan–Meier curves to describe mortality and disease-free survival in each group, with log-rank tests used for between-group comparisons. Multivariable Cox regression was applied to study the association between chemotherapy use and patient survival while controlling for potential confounders. Variables that were significantly different between groups were considered potential confounders. All statistical tests were two-sided, with *p* < 0.05 considered statistically significant. The analyses were performed using SPSS software (IBM SPSS version 28, Armonk, NY, USA, 2021).

## 3. Results

**Figure 2:**
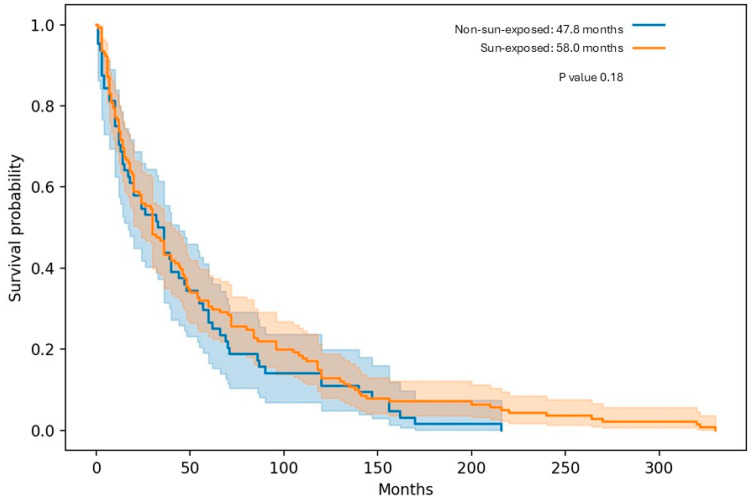
Disease-Free Survival for sun-exposed vs. non-sun-exposed groups.

In our archives, we identified 249 patients from a multicenter cohort in Israel who were diagnosed with MCC local or locally advanced disease between January 1985 till December 2020. Of these, 147 (59%) had tumors in sun-exposed areas, 84 (33.8%) non-sun-exposed areas, and 18 (7.2%) had MCC of unknown primary. The cohort comprised 152 (61.05%) males and 97 (38.95%) females. Of them 225 patients met the eligibility criteria and had all the relevant information for this study and were included in the analysis (Figure 2). Of those, 142 patients were categorized under sun-exposed areas (cohort A) and 83 under non-sun-exposed areas (cohort B). Each cohort was arranged for age at diagnosis, gender, primary tumor (T) diameter (less or over 2 cm), LN involved or not involved at diagnosis, DFS and OS.

### 3.1. Demographic Characterization of the Cohorts

#### 3.1.1. Sun-Exposed

Cohort A included 142 patients with sun-exposed primary tumors. The cohort comprised 85 males (60.0%) and 57 females (40.0%). The median age at diagnosis was 73 years (range 37–96). Among males, the median age was 72.7 years (range 41–96), while among females it was 73.5 years (range 37–94). Lymph node involvement was identified in 43 patients (30.0%), whereas 99 patients (70.0%) had no nodal involvement. With respect to primary tumor size, 69 tumors (48.6%) measured <2 cm, 66 tumors (46.4%) measured ≥2 cm, and tumor diameter was unspecified (Tx) in 7 cases (5.0%) (Table 1).

#### 3.1.2. Non-Sun-Exposed

Cohort B comprised 83 patients with non-sun-exposed primary tumors. The cohort included 55 males (66.3%) and 28 females (33.7%). The median age at diagnosis was 72.6 years (range 48–96). Among males, the median age was 72.4 years (range 56–96), while among females it was 73.1 years (range 48–91).

Lymph node involvement was observed in 47 patients (57.0%), whereas 36 patients (43.0%) had no evidence of nodal involvement. Regarding primary tumor size, 45 tumors (54.2%) measured <2 cm, 36 tumors (43.4%) measured ≥2 cm, and tumor diameter was unspecified (Tx) in 2 cases (2.4%) (Table 1).

### 3.2. Survival Outcomes According to Sun Exposure Status

In the overall cohort, median DFS was numerically longer in Sun-Exposed patients compared with Non-Sun-Exposed patients (58.0 vs. 47.8 months), although this difference did not reach statistical significance in our analysis (*p* ≈ 0.18) (Table 2 and Figure 2).

**Figure 3:**
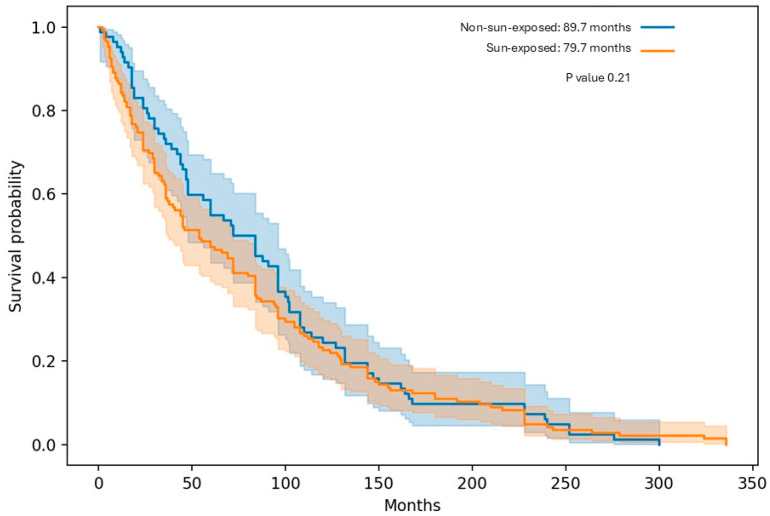
Overall survival for sun-exposed vs. non-sun-exposed groups.

Similarly, median OS showed a numerical advantage for Non-Sun-Exposed patients (89.7 vs. 79.7 months), without a statistically significant difference (*p* ≈ 0.21), (Table 3), (Figure 3). When stratified by gender, females demonstrated longer DFS and OS than males in both exposure groups. Among females, DFS appeared longer in the Sun-Exposed group compared with the Non-Sun-Exposed group (70.8 vs. 53.5 months, *p* ≈ 0.12), while OS numerically favored Non-Sun-Exposed females (99.0 vs. 87.6 months, *p* ≈ 0.15). No significant differences were observed among male patients for either DFS or OS.

Notably, lymph node–negative patients demonstrated improved outcomes compared with node-positive patients across both exposure groups. In the LN-negative subgroup, Non-Sun-Exposed patients showed a significantly longer OS compared with Sun-Exposed patients (105.9 vs. 91.4 months, *p* ≈ 0.03). This difference was particularly evident among LN-negative males (101.4 vs. 81.6 months, *p* ≈ 0.04), while a similar trend was observed in females without reaching statistical significance (*p* ≈ 0.07). In contrast, no meaningful DFS or OS differences were observed between exposure groups among patients with LN involvement at diagnosis. Primary tumor size further stratified outcomes. Among patients with tumors <2 cm, Non-Sun-Exposed patients experienced significantly longer OS compared with Sun-Exposed patients (111.7 vs. 91.9 months, *p* ≈ 0.02), with this benefit observed in both males and females. Conversely, among patients with tumors ≥2 cm, Non-Sun-Exposed patients demonstrated significantly longer DFS (47.5 vs. 35.1 months, *p* ≈ 0.03) and OS (76.5 vs. 53.3 months, *p* ≈ 0.01) compared with Sun-Exposed patients, a pattern that was consistent across genders.

In a multivariable logistic regression model adjusting for age at diagnosis, TNM stage, and tumor location, chemoradiotherapy was not significantly associated with mortality at either 5 years (HR = 1.547, 95% CI 0.656–3.65, *p* = 0.319) or 20 years (HR = 1.358, 95% CI 0.614–3.003, *p* = 0.450). In contrast, age at diagnosis remained significantly associated with overall survival (Table 4).

## 4. Discussion

In the current multicenter real-world retrospective study we aim to verify the outcomes of the epidemiology and pathogenesis of MCC in Israeli patients. The primary focus is to investigate whether the area of the tumor’s origin among our patients, particularly concerning exposure or lack of exposure to sunlight, plays a role in influencing the tumor’s recurrence or overall survival. Furthermore, we aim to analyze how subgroups based on factors such as gender, whether lymph nodes are involved or not at diagnosis, and tumor size (less or more than 2 cm) might be affected by sun exposure or lack thereof.

MCC is known to be a very rare disease with an incidence rate below 1%, ranging from 0.5% to 0.7% cases per 100,000 people worldwide [1,2,3]. Over the last three decades, there has been a consistent increase in the incidence of MCC. Examination of the Surveillance, Epidemiology, and End Results registry in the United States unveiled a rate of 0.79 cases per 100,000 person-years in 2011, indicating roughly 1600 new cases annually. This marks a substantial 95% annual surge since 2000. Similarly, European countries, Australia, and China have witnessed a growing incidence of MCC over the past few decades, while in Israel according to estimates, about 40 new MCC cases are discovered per year, of which about 12–15 are metastatic [4,17].

There are two main causes linked to MCC. The first revolves around MCPyV infection, which is not uncommon. MCC development requires not only infection but also integration into the tumor genomes and a specific mutation in the MCPyV large T-antigen, rendering it incapable of replication. These MCPyV-derived oncoproteins trigger various immune responses, with reported positivity rates of 70–80%, primarily documented in North America and Europe. However, MCPyV positivity rates exhibit significant geographical discrepancies. For instance, while Australia sees an MCC incidence approximately double that of North America and Europe, its MCPyV positivity rate is only around 20%. Conversely, Asia experiences lower MCC incidences compared to Western regions, yet in a Japanese cohort of 71 patients, the MCPyV positivity rate was notably high at 69%. This discrepancy ties not just to sun exposure and skin color variations but also to differences in the MCPyV genotype itself [18,19,20,21,22,23,24,25].

The other primary cause is linked to sun exposure, particularly concerning MCPyV-negative MCCs, where genetic damage induced by UV radiation serves as the oncogenic trigger. Tumors in these cases develop through disruptions in the Rb and p53 tumor suppressor pathways, albeit via different mechanisms. Recurring inactivating mutations in the TP53 and RB1 genes drive the process of oncogenesis. For instance, in regions like Australia, UV radiation stands out as the predominant etiopathogenetic factor contributing to MCC development [23,26,27,28]. As we have shown, more patients with sun-exposed areas (174 patients) than non-sun-exposed areas (84 patients) were diagnosed with MCC in Israel between the years 1985–2020.

Upon comparing our findings with established literature, we observed a higher incidence of MCC in males compared to females, typically at a ratio of 2:1. This gender discrepancy aligns with our patient data. Additionally, MCC tends to be most prevalent among individuals aged over 70 years, a trend consistent with our own results [27,28,29,30]. Sentinel lymph node biopsy stands as the most dependable staging method for uncovering subclinical nodal disease. According to established literature, approximately 30% and up to 50% of patients with primary MCC exhibit subclinical node metastases [31]. However, our subgroup analysis revealed intriguing insights. In cohort A (Sun-Exposed sites), 30% of patients displayed LN involvement at diagnosis which is compatible with known. Conversely, in cohort B (Non Sun-Exposed sites), a higher proportion, specifically 57% of patients, demonstrated LN involvement, surpassing the figures documented in existing literature and double than cohort A.

Additionally, MCC tends to be most prevalent among individuals aged over 70 years, a trend consistent with our own results [26,27,28]. Sentinel lymph node biopsy remains the most dependable staging method for detecting subclinical nodal disease. According to established literature, approximately 30% to 50% of patients with primary MCC exhibit subclinical lymph node metastases [29]. In our subgroup analysis, this distribution differed notably by sun exposure status. In cohort A (sun-exposed areas), 30.0% of patients demonstrated lymph node involvement at diagnosis, consistent with published data. In contrast, cohort B (non–sun-exposed areas) showed a substantially higher rate of lymph node involvement, affecting 57.0% of patients, which exceeded reported rates in the literature and was nearly double that observed in cohort A (Table 1).

When comparing MCC outcomes between cohort A and cohort B, median mDFS was lower in cohort B, with a median of 47.8 months, compared with 58.0 months in cohort A. In contrast, median OS was higher in cohort B, reaching approximately 89.7 months, compared with 79.7 months in cohort A. Regarding gender, both cohorts demonstrated a consistent trend in which female patients exhibited longer survival outcomes than males for both mDFS and mOS.

In the overall cohort, median DFS was numerically longer in Sun-Exposed patients compared with Non-Sun-Exposed patients (58.0 vs. 47.8 months), and Similarly, median OS showed a numerical advantage for Non-Sun-Exposed patients (89.7 vs. 79.7 months), in which may be explained by a several biological mechanisms may underlie the observed and consistent prognostic differences. One of the most notable factors is the variation in MCPyV status according to anatomical site [29]. Evidence indicates that MCPyV prevalence differs significantly by tumor location. For example, only 46.2% of head and neck MCC cases were found to be MCPyV-positive, compared with 92.3% of tumors arising on the trunk and extremities (*p* = 0.003) [30]. Multiple studies have confirmed this pattern, demonstrating that virus-positive tumors are more frequently located on the extremities, whereas sun-exposed regions—particularly the head and neck—show a predominance of virus-negative disease. The clinical relevance of this distribution becomes evident when outcomes are considered. MCPyV-negative tumors consistently exhibit more aggressive behavior. This is largely attributed to their substantially higher tumor mutational burden, nearly 100-fold greater than that observed in virus-positive tumors, reflecting extensive UV induced DNA damage [29,30,31,32]. These tumors display characteristic UV-associated mutational signatures and genomic instability. In addition, virus-negative MCC commonly harbors TP53 and RB1 mutations, while virus-positive tumors are primarily driven by viral oncoproteins, resulting in distinct oncogenic pathways. Although the clinical presentation may be similar, these divergent molecular mechanisms give rise to fundamentally different tumor biology [30,31,32].

Another contributing factor may involve the local immune environment. Chronically sun-exposed skin undergoes long-term UV-induced immunosuppression, which can impair immune surveillance and facilitate tumor immune evasion. This sustained alteration of the cutaneous immune milieu may create a permissive microenvironment that supports more aggressive tumor growth, such as in the head and neck region [31,32,33,34,35].

With respect to lymph node involvement at diagnosis, in cohort A, lymph node positivity was associated with shorter mDFS and mOS compared with patients without nodal involvement. In cohort B, lymph node involvement similarly corresponded to reduced mDFS; however, mOS was generally higher than in cohort A, particularly among female patients. Among patients without lymph node involvement at diagnosis, both cohorts demonstrated improved survival outcomes. Cohort A showed a slightly longer mDFS compared with cohort B, whereas cohort B exhibited significantly longer mOS, especially among male patients.

Tumor size also influenced survival outcomes in both cohorts. Patients with primary tumors smaller than 2 cm experienced more favorable outcomes than those with tumors ≥2 cm. For tumors <2 cm, cohort A demonstrated longer mDFS, while cohort B showed significantly longer mOS, particularly among female patients. Conversely, in tumors ≥2 cm, cohort B consistently exhibited longer mDFS and mOS compared with cohort A across both sexes. Overall, these findings suggest that survival outcomes in MCC vary meaningfully by sun exposure status, sex, lymph node involvement, and tumor size, with female sex, absence of nodal disease, and smaller tumor size consistently associated with improved survival.

Noticing that regions with sun exposure show worse outcomes compared to non-sun-exposed areas raises awareness regarding the potential adverse impact of excessive sunlight exposure on our patients’ survival. Additionally, emphasizing earlier diagnosis appears correlated with improved survival rates.

While our study yields valuable insights into the contrasting outcomes of Merkel Cell Carcinoma in Sun-Exposed Areas versus Non-Sunlight Exposure, we must acknowledge several limitations. The retrospective data sourced from multiple institutions spanning different periods, authored by various physicians, may introduce potential biases. However, it’s crucial to note that data collection was standardized by a single person. The study’s small size, albeit reflective of the rarity of this disease, poses limitations. Additionally, the unavailability of MCPyV status assessment presents a constraint. Future investigations should explore alternative pathogeneses and conduct comparative analyses to elucidate their impact.

To fortify these findings, future studies should encompass diverse institutions or nations, engaging more extensive patient cohorts. Despite our study’s limitations, it’s essential to underscore the rarity of MCC, particularly in Israel, with nearly 40 cases annually of them approximately 15 in a metastasized stage. This study marks, to the best of our knowledge, the initial national comparison between Sun-Exposed Areas and Non-Sunlight Exposure in Merkel Cell Carcinoma. Our findings hold substantial implications for managing and monitoring this patient population.

## 5. Conclusions

In our real-world cohort, we provide important evidence demonstrating distinct clinical patterns and outcome differences associated with primary tumor location in sun-exposed versus non-sun-exposed areas among patients diagnosed with MCC. Moreover, our study further examines clinically relevant subgroups stratified by sex, lymph node involvement at diagnosis, and primary tumor size. We evaluated the impact of sun exposure status within these subgroups on median DFS and median OS, comparing patients with tumors arising in sun-exposed areas to those with tumors located in non-sun-exposed regions.

## Figures and Tables

**Table 1:** Baseline Characteristics According to Sun Exposure Status.

Characteristic	Sun-Exposed (*n* = 142)	Non-Sun-Exposed (*n* = 83)	*p* Value
**Gender**			0.42
Male	85 (60.0%)	55 (66.3%)	
Female	57 (40.0%)	28 (33.7%)	
**Age at diagnosis, years**	73 [37–96]	72.6 [48–96]	NA
Male	72.7 [41–96]	72.4 [56–96]	
Female	73.5 [37–94]	73.1 [48–91]	
**LN involvement**			<0.001
Yes	43 (30.0%)	47 (57.0%)	
No	99 (70.0%)	36 (43.0%)	
**Primary tumor (T) diameter**			0.53
<2 cm	69 (48.6%)	45 (54.2%)	
≥2 cm	66 (46.4%)	36 (43.4%)	
Tx (unspecified)	7 (5.0%)	2 (2.4%)	

**Table 2:** Disease-Free Survival Outcomes by Sun Exposure.

Characteristic	Sun-Exposed mDFS (Months)	Non-Sun-Exposed mDFS (Months)	*p* Value
**Overall**	58.0	47.8	0.18
**Gender**			
Male	49.8	45.0	0.34
Female	70.8	53.5	0.12
**LN involved at diagnosis**			
Overall	50.2	45.3	0.41
Male	46.4	37.9	0.28
Female	54.6	63.5	0.22
**LN not involved at diagnosis**			
Overall	65.2	60.8	0.39
Male	56.7	63.2	0.31
Female	72.5	48.6	0.04
**Primary tumor < 2 cm**			
Overall	71.6	67.1	0.45
Male	54.8	57.6	0.62
Female	87.0	76.5	0.21
**Primary tumor ≥ 2 cm**			
Overall	35.1	47.5	0.03
Male	41.4	44.7	0.47
Female	36.1	52.4	0.04

Abbreviations: cm, Centimeter; mDFS, Median Disease-Free Survival; LN, Lymph nodes.

**Table 3:** Overall Survival based on Sun Exposure.

Characteristic	Sun-Exposed mOS (Months)	Non-Sun-Exposed mOS (Months)	*p* Value
**Overall**	79.7	89.7	0.21
**Gender**			
Male	74.3	81.9	0.27
Female	87.6	99.0	0.15
**LN involved at diagnosis**			
Overall	76.3	81.2	0.38
Male	76.4	77.1	0.91
Female	75.8	91.9	0.04
**LN not involved at diagnosis**			
Overall	91.4	105.9	0.03
Male	81.6	101.4	0.04
Female	100.0	115.0	0.07
**Primary tumor < 2 cm**			
Overall	91.9	111.7	0.02
Male	82.5	95.5	0.08
Female	105.8	128.2	0.03
**Primary tumor ≥ 2 cm**			
Overall	53.3	76.5	0.01
Male	57.4	78.4	0.03
Female	47.2	83.7	0.01

Abbreviations: cm, Centimeter; mOS, Median Overall Survival; LN, Lymph nodes.

**Table 4:** Multivariate Analysis of Factors Associated with 5-Year and 20-Year Overall Survival.

OS-5 Years			OS-20 Years			
Variable	HR	95% CI	*p* Value	HR	95% CI	*p* Value
Chemotherapy as adjuvant therapy	1.547	0.656–3.65	0.319	1.358	0.614–3.003	0.45
Age at diagnosis	1.064	1.029–1.100	<0.001	1.069	1.039–1.101	<0.001
TNM stage			0.696			0.722
I	Ref *			Ref *		
II	1.114	0.385–3.229		0.696	0.283–1.713	
III	1.492	0.570–3.904		0.827	0.373–1.833	

Abbreviations: *—Reference group.

## Data Availability

The data presented in this study are available on request from the corresponding authors.

## Abbreviations

The following abbreviations are used in this manuscript:

MCC	Merkel cell carcinoma
LN	Lymph node
DFS	Disease-free survival
OS	Overall survival
MCPyV	Merkel cell polyomavirus
CT	Computed tomography
FDG	Fluorodeoxyglucose
PET-CT	Positron emission tomography computed tomography
NCCN	National Comprehensive Cancer Network
UV	Ultraviolet
mDFS	Median disease-free survival
